# Health status and self-perception of health among homeless people in Spain: a mixed-methods study

**DOI:** 10.3389/fpubh.2024.1444888

**Published:** 2024-08-29

**Authors:** Miguel A. Bedmar, Laura Capitán-Moyano, Miquel Bennasar-Veny, Cristina Moreno-Mulet, Alba Carrero-Planells, Aina M. Yáñez

**Affiliations:** ^1^Research Group on Global Health and Human Development, University of the Balearic Islands, Palma, Spain; ^2^Department of Nursing and Physiotherapy, University of the Balearic Islands, Palma, Spain; ^3^Health Research Institute of the Balearic Islands (IdISBa), Palma, Spain; ^4^CIBER de Epidemiología y Salud Pública (CIBERESP), Institute of Health Carlos III, Madrid, Spain; ^5^Qualitative and Critical Health Research Group, University of the Balearic Islands, Palma, Spain; ^6^Research Network on Chronicity, Primary Care, and Health Promotion (RICAPPS), Institute of Health Carlos III, Madrid, Spain

**Keywords:** homelessness, social justice, employment, social exclusion, health inequities, social determinants of health

## Abstract

**Introduction:**

Homelessness is a phenomenon of social exclusion and poverty that has increased alarmingly during recent years. Homeless people (HP) experience violations of several basic human rights or needs that impact their well-being. Therefore, the aim of this research was to assess the health status and self-perceived health of HP and examining the impact of homelessness on their health.

**Methods:**

We used an explanatory sequential mixed-methods approach that integrated a quantitative cross-sectional study within critical social and ethno-sociological qualitative frameworks. Data were collected in Palma, Spain, from December 1, 2020, to January 1, 2023. A total of 198 HP were recruited from the streets and public areas. Basic human needs (Virginia Henderson model), medical diagnoses, substance abuse (DAST-10), diet quality (IASE), depression (PHQ-9), and social support (SSQ-6) were assessed. Then, 17 semi-structured interviews were conducted and were analyzed using thematic content and discourse analysis. Quantitative and qualitative data were integrated and jointly analyzed.

**Results:**

The 79.3% of the participants were men, mean age of 47.8 ± 12.2 years. The 76.4% were Spanish. The participants reported severe difficulties in accessing the labor market, and that this greatly affected their self-esteem and mental health. The 48.9% of the participants suffered from one or more chronic diseases, and 50.3% were diagnosed with a mental health disorder. The participants generally did not consider health problems as a central concern. The health needs that most affected the participants were related to food, safety, and social support. HP frequently felt unsafe, fearfully, and anxious.

**Conclusion:**

Homelessness, unemployment, and social exclusion have significant negative impacts on the health and wellbeing of HP. Precarious work conditions and deficiencies in the welfare system contribute significantly to homelessness. These results highlight the need for systemic solutions that extend beyond short-term housing initiatives.

## Introduction

Homelessness has increased considerably in Western countries over the last decade (1). In Europe, at least 895,000 individuals live on the streets or in public spaces, spend nights in emergency shelters, or are accommodated in temporary facilities (2). Additionally, 21.6% of all Europeans (95.3 million people) are at risk of poverty or social exclusion (3). Spain ranks as is the third-worst among European countries (26%) on the At-Risk-Of-Poverty or Social Exclusion (AROPE) indicator (4). Specifically, 28,552 people in Spain are affected by homelessness, a 24.5% increase since 2012 (5). However, these data on housing exclusion only consider the most visible and severe cases, and do not include other situations related to housing insecurity (2).

The increase of homelessness can be attributed to world-wide social crises, armed conflicts, climate emergencies, and immigration, factors that are complex and interrelated (6). However, unaffordable housing is the central cause of all forms of homelessness. Between 2010 and 2022, housing and rental prices have increased in Spain, mirroring the trends throughout Europe (3). The transformation of housing into a speculative investment is a significant driver of this increase (7).

Housing is recognized as fundamental human right by international treaties and the Sustainable Development Goals (SDGs), and is considered pivotal for achieving physical, psychological, and social well-being (8). In response, the European Federation of National Organizations Working with the Homeless (FEANTSA) currently advocates for several legislative measures, such as regulation of the real estate market, prevention of forced evictions, and promoting public housing for vulnerable populations (2).

The consequences of homelessness are multi-dimensional, and social exclusion is one of the major effects. Housing deprivation extends beyond the purely physical dimension, because it can lead to the progressive loss of an individual’s affective, social, and personal niche (9). Homeless people (HP) have increased rates of mortality and morbidity (10, 11) that can be attributed to substantial health inequities (12, 13). For example, approximately 80% of HP struggle with mental health issues (5), about half of them have an elevated risk of suicide (14), and about half of them suffer from chronic diseases that affect the cardiovascular or respiratory systems (15). Chronic physical diseases in HP account for about two-thirds of the cases of premature mortality (16).

Homeless populations are highly susceptible to violence, and one study reported that three-quarters of them were victims of violence (5). Homeless women in particular face alarming rates of physical, psychological, and sexual assault (17, 18). The decreased accessibility of healthcare services and the difficult living conditions experienced by HP play pivotal roles in determining their health outcomes (19). The complexity of comorbidities and severe mental illnesses among HP (20), coupled with the lack of continuity and palliative care (21), pose significant challenges for healthcare systems.

Although the scientific community has quantified the number of HP, analyzed the economic causes of homelessness, and examined the prevalence of different health conditions among HP, there is comparatively little known about their self-perceived well-being, needs, and health.

## Theoretical framework

As shown by previous studies (22) and advocated by Nussbaum and Sen (23), the theoretical approaches to studying homelessness often use the perspective of social justice. Sen (24) proposes the “capability approach” to address social justice in homelessness, since this approach recognizes that different individuals have different opportunities, resources, and freedoms to develop “doings and beings.” Nussbaum (25) emphasizes the role of capabilities in constructing a normative conception of social justice by specifying a specific set of capabilities that need the most protection. According to Nussbaum (26), the development of well-being is interconnected with the development of health. This understanding of the connection between well-being and health depends on an individual’s aspirations for a particular lifestyle, and on the individual’s preferences and priorities. Therefore, examination of the impact of homelessness on health requires study of the fundamental aspects that are valued by each person. The “capability empowerment” approach represents a rights-based evaluative approach that originated from studies of poverty and development.

However, Nussbaum’s capabilities theory has been criticized for its individualistic approach, because this approach may not adequately address the complexity of homelessness (27). Therefore, we combined the theoretical framework of the current study with a critical perspective that is based on the Social Determinants of Health (SDOH) approach (28) to better understand the relationship between homelessness and health (29). This approach explains the reasons for getting sick within a specific socioeconomic and political context and recognizes that this context generates an unequal power relationship that affects the number of opportunities. Specifically, the SDOH approach considers structural factors, such as employment, housing accessibility, political measures, healthcare systems, and gender to better understand the health of HP.

Finally, after exercising reflexivity and positionality, and following a defined theoretical approach, our research team faced the phenomenon of study as an active exercise of health advocacy (30, 31). This viewpoint considers the scientific community and professionals as groups that fight against social and health inequities. Therefore, we perceive homelessness as something that is avoidable, unfair, and repairable that requires a deep analysis from each individual’s point of view and includes consideration of the context (26, 32).

## Methods

The aim of this study was to assess the health status and self-perceived health of individuals experiencing homelessness and to explore the effect of homelessness on health. We used an explanatory sequential mixed-methods approach (quan-QUAL) (33). Mixed-methods research allows exploration of divergent viewpoints on the same issue and provides contextual understandings that are shaped by real-life experiences and cultural influences (34). In particular, we used a cross-sectional and ethno-methodological design that included critical discourse analysis.

### Quantitative design

#### Participants

We aimed to reach all potentially HP in Palma, Spain (approximately 300 individuals according to 2019 data) (35). HP were defined as those living in the streets or other public areas, in an abandoned building, or in places that did not meet the minimum conditions for habitability. Notably, within the same week or month, a HP could live in the street, spend several nights in a municipal shelter, spend another week in the makeshift shelter of a friend, and then return to the streets. This approach considers homelessness as the continuous instability of an overnight location and classifies most participants in the “Roofless” category according to the ETHOS classification (36). Thus, if a person slept in an abandoned building or a makeshift shelter at the moment of recruitment, they were still considered as “Roofless” (although they would be in the “inadequate” or “insecure” conceptual category in the ETHOS classification). This classification is based on the perception of homelessness as a continuous (not a static) phenomenon.

Inclusion criteria were homelessness and age of at least 18 years. Exclusion criteria were staying overnight in a private or municipal shelter for more than 3 months (in contrast to housing instability), experiencing an acute mental health episode, or inability to answer the questionnaire.

#### Data collection and recruitment

We used an investigator-administered questionnaire that examined basic needs (an adaptation of Virginia Henderson’s approach to the specific needs of homeless individuals) (37), substance abuse (DAST-10, Drug Abuse Screening TEST), diet quality (IASE, Healthy Eating Index), depressive symptoms (PHQ-9, Patient Health Questionnaire), and perceived social support (SSQ-6, Social Support Questionnaire). Health data were obtained from the electronic medical records of the Health Service of the Balearic Islands. Data on the use of health care services and hospital admissions from 2019 to 2021 were retrieved from the PRISIB (the research platform on health information of the Balearic Islands). All participants were recruited in collaboration with different organizations that assist HP (Spanish Red Cross and Medicos del Mundo NGOs and public social service IMAS).

Surveys were conducted between December 2020 and September 2021 and lasted 20–40 min. Financial compensation was offered to all participants. Further details of the data collection procedures were provided elsewhere (38).

#### Data analysis

Continuous variables are presented as medians and interquartile ranges (IQR) because statistical normality could not be assumed based on the Kolmogorov–Smirnov test. Categorical variables are presented as absolute numbers and percentages. All statistical analyses were conducted using IBM SPSS Statistics version 26 (SPSS/IBM, Chicago, Illinois, United States).

### Qualitative design

#### Participants

Seventeen HP (5 women, 12 men) participated in the qualitative phase that were segmented according to gender, living conditions, age, and place of birth. The aim of this phase was to achieve the most significant heterogeneity participant discourse. We followed established criteria to set the sample size (39). These criteria are based on the “information power” of a sample, which depends on (a) the aim of the study, (b) sample specificity, (c) use of established theory, (d) quality of dialogue, and (e) analysis strategy.

Participants were selected using theoretical and purposive sampling according to their ability to provide a narrative that described their homelessness. Snowball sampling was used to select additional subjects. Key informants at social or homeless organizations recruited participants, and the main investigator (MAB, who has worked as a nurse in a shelter for HP), contributed to the recruitment process.

#### Data collection and recruitment

After initial analysis of data in the quantitative phase, semi-structured interviews were conducted (40). These data were collected using semi-structured interviews performed by MAB. To prevent the possible effects of power relationships during the interview, MAB did not reveal his background to the participants. All questions were inspired by the previously described theoretical frameworks, and covered perceived health status, access and use of health and social resources, the social environment, perceived social support, and perception of vulnerability and safety. The life history of each participant and its impact on homelessness was also analyzed.

Interviews were conducted between December 1, 2021 and January 1, 2023. We initially conducted three interviews, and then comprehensively analyzed the data collection and interview processes to refine the interview structure and the representativeness of participants’ profile. The discourse analysis was performed simultaneously to data collection. Finally, after 17 interviews, the data were saturated.

The interviews were conducted in a private room of the night shelter facilities of an NGO. All interviews lasted approximately 60–90 min and were audio-recorded, and then transcribed. We offered financial compensation to all participants. A more detailed description of the data collection procedures was provided elsewhere (38). The lead researcher (MAB) also recorded field notes during and after all interviews.

#### Data analysis

An abductive content and critical discourse analysis were carried out. Critical discourse analysis studies the ways in which social power abuse, dominance, and inequality are enacted, reproduced, and resisted, and focuses on communication by text and speech in the social and political contexts. This type of analysis seeks to understand, expose, and ultimately overcome social inequality (41).

The data analysis consisted of the following three steps: (I) familiarization with the data by repeated reading of the transcribed interviews; (ii) identification of codes and categories by inductive and deductive strategies; and (iii) analysis and interpretation according to the conceptual framework (SDOHs, and Nussbaum’s capabilities). Data were codified and analyzed independently by three researchers. Triangulation was then performed to compare codification, identify convergences and divergences, and reach a final consensus. Field notes were integrated into the interview transcripts and were also used in the analysis. A pseudonym for each participant was used to preserve the anonymity. The data analysis was systematized and optimized with ATLAS.ti software (version 9.0).

#### Scientific rigor

Techniques, such as saturation of the discourse, triangulation among researchers, data collection techniques, and theoretical frameworks were employed to ensure methodological rigor and validity (42). Reflexivity of the principal investigator was used throughout the research process by field diary (43).

#### Integrating quantitative and qualitative data

The study design provided integration by use of an explanatory sequential design. Thus, the quantitative data were first gathered and analyzed (Phase 1), and these results were used to inform the collection of qualitative data (Phase 2). For integration at the methodological level, we used the following two approaches: (I) integration by “connecting” (connecting samples; some participants from Phase 1 continued to participate in Phase 2); and (ii) integration by “building” (results from the qualitative phase provided more insight and closed research gaps) (41, 44). Integration at the interpretation and reporting levels was provided by using the “weaving approach,” in which data from the quantitative and qualitative phases were reported side-by-side in narrative form (41, 44). This approach was used to identify consistencies and inconsistencies of the quantitative and qualitative data, and to determine when the qualitative data expanded the findings of the quantitative data, or vice versa. Finally, we used a joint display table to integrate and display all results (41).

## Results

We included 198 HP in the study (mean age: 47.8 ± 12.3 years; 79.3% males; Table 1), most of whom (76.5%) were born in Spain. A total of 29.8% of the participants had education below the level of secondary school, and the duration of homelessness was highly variable (median: 1 year, IQR: 4 years). More than half of the participants (81.9%) lived in public spaces or night shelters, and the others lived in abandoned buildings or similar places. The participants in the quantitative and qualitative phases had similar sociodemographic characteristics.

**Table 1:** Sociodemographic characteristics of the study population.

	Quantitative overall sample *n* = 198 *n* (%)	Qualitative overall sample *n* = 17 *n* (%)
Gender identity—male	157 (79.3%)	12 (70.6%)
Age (years)—mean (SD)	47.81 (±12.3)	–
<30	15 (7.6%)	–
30–39 40–49 50–59 ≥ 60	38 (19.2%) 58 (29.3%) 47 (23.7%) 40 (20.2%)	3 (17.6%) 7 (41.2%) 5 (29.4%) 2 (11.8%)
Educational level		
Less than elementary school	11 (5.6%)	2 (11.8%)
Elementary school	48 (24.2%)	8 (47.0%)
Secondary school	84 (42.4%)	4 (23.5%)
Specific vocational training	27 (13.6%)	1 (5.9%)
University degree	14 (7.1%)	1 (5.9%)
N/A	14 (7.1%)	–
Country of birth - Spain	118 (59.6%)	13 (76.5%)
Duration of homelessness (*n* = 192)^a^		
Less than 1 year	69 (35.9%)	8 (47.0%)
Between 1 and 3 years	63 (32.8%)	5 (29.4%)
More than 3 years	60 (31.3%)	5 (29.4%)
Overnight place (*n* = 195) Roofless		
Public space	111 (56.8%)	6 (35.3%)
Night shelter	49 (25.1%)	7 (35.3%)
Illegal occupation (*abandoned building or makeshift shelter*)	35 (17.9%)	4 (23.5%)

a This refers to how long an individual had been homeless or in residential exclusion. We distribute the duration of homelessness around the COVID-19 crisis to try to estimate the impact of it on homelessness.

### Underground economy and unemployment

The 91.4% were unemployed and only 2 participants were legally employed (Table 2). The most important barriers to employment were health issues (27.3%), the COVID-19 crisis (25.7%), old age (12.6%), and undocumented immigrant status (12.1%). The last reported employment contract was an average of 5 years ago (before the onset of the COVID-19 pandemic).

**Table 2:** Employment and access to the labor market.

	Quantitative overall sample *n* = 198 *n (%)*
Employment	
Employed (legal economy)	2 (1.0%)
Unemployed	181 (91.4%)
Retired	9 (4.5%)
NA	6 (3.0%)
Unemployment benefit^a^—No	136 (68.7%)
Last employment contract	
Time since last employment contract—mean (SD)	4.9 (6.5)
Difficulties in accessing the labor market^b^	
Health issues	54 (27.3%)
COVID-19 crisis	51 (25.7%)
Old age	25 (12.6%)
Illegal migration	24 (12.1%)

a Unemployment insurance or social welfare benefit (post-release economic support, basic economic subsidies, etc.).

b Self-reported.

Participants explained that health problems and the very physically demanding tasks typical of precarious jobs (e.g., construction or cleaning) prevented them from entering or continuing in legal employment. Furthermore, they reported job loss due to workplace accidents, aging, or chronic diseases.

*“I work in construction, and when I was working, blood came out (rectal hemorrhage), and the doctor told me to not lift heavy weights. I left construction, but there was no other job. I didn’t know how to find anything else.” (Emmanuel, man, makeshift shelter)*

Nearly 70% of the unemployed participants did not receive unemployment benefits from the Spanish public welfare system, and they explained the challenges encountered when seeking these benefits. They said that certain very strict requirements, such as the need to present a census certificate or have a legal administrative status, prevented access to these benefits. One participant mentioned the difficulties in getting social benefits due to his young age.

*“Until you are 45 years old you have no rights to get any social benefit. (Antonio, man, abandoned building)*

Even those who received unemployment benefits said that this financial support was insufficient for securing a permanent residence or purchasing food and other essential items, causing them to remain on the streets or in shelters.

On the other hand, the participants reported a heavy reliance on the underground economy as the sole source of income. These opportunities were limited to begging, scavenging for scrap materials (primarily men), engaging in cleaning tasks, and prostitution (primarily women).

*“I worked so hard since I arrived in Spain, cleaning. Then, in a beach bar, I clean it, I do the shopping for them, or I decorate it for Christmas, and that’s how I earn money… Even prostitution” (Andrea, woman, abandoned building)*

The lack of employment and the conditions at precarious jobs have serious emotional impacts on HP. Participants reported that unemployment, particularly over an extended period, had serious implications. They emphasized that employment provides financial stability, gives them social status and a sense of community, and these affect their identity and dignity.

*“Well, I would also like to have a permanent job, or even if it is temporary, whatever … But having a job, that is what all normal people do” (Francisca, woman, abandoned building)*

### Health concerns among HP

Approximately half of the participants (87/179, 48.9%) were diagnosed with one or more chronic diseases (Table 3), and liver diseases were the most prevalent (39/87, 44.8%) Other participants had high blood pressure (28/87, 32.2%), cardiovascular pathologies (14/87, 16.1%), COPD (11/87, 12.6%), asthma (10/87, 11.5%), and type 2 diabetes mellitus (10/87, 11.5%).

**Table 3:** Physical and mental health conditions.

	Quantitative overall sample *n* = 179 *n (%)*
Physical chronic diseases	87 (48.9%)
Liver disease	39 (21.8%)
High blood pressure	28 (15.6%)
Cardiovascular pathologies	14 (7.9%)
Chronic obstructive pulmonary disease	11 (6.1%)
Asthma	10 (5.6%)
Diabetes mellitus type 2	10 (5.6%)
Chronic kidney disease	2 (1.1%)
Infectious diseases	54 (30.2%)
Hepatitis C	39 (21.8%)
HIV^a^	12 (6.7%)
Syphilis	9 (5.0%)
Hepatitis B	6 (3.3%)
Mental health disorders	90 (50.3%)
Mood disorder	86 (48.0%)
Symptoms of major depressive disorder^b^	74 (41.3%)
Suicide attempt	7 (3.9%)
Substance use disorder (*n* = 173)^c^	108 (54.5%)
Schizoaffective disorder	7 (3.9%)
Trimorbidity^d^	26 (14.5%)

a None of those with HIV had AIDS.

b Data from PHQ-9.

c Data from health substance abuse services, DAST-10 and diagnoses from medical records.

d Mental health disorder (including drug abuse), physical chronic disease and infectious disease.

A total of 30.2% of the participants (54/179) presented with a communicable disease, and 72.2% of these individuals (39/54) had hepatitis C, and 22.2% (12/54) had HIV, although none had AIDS.

More than half of the participants (90/179, 50.3%) were diagnosed with a mental health disorder. Among these 90 participants, mood disorders were the most prevalent (86, 95.6%), 7 participants (3.9%) had schizoaffective disorder, and 74 participants (41.3%) had symptoms of depression according to the PHQ-9. Overall, 108 of 173 participants (54.5%) had substance abuse disorders, although we did not identify the specific substances. Moreover, 26 of 179 participants (14.5%) suffered from trimorbidity (physical health, mental health, substance abuse) which included communicable diseases.

Although multiple physical and mental health disorders were common among the participants, and they refer it has an impact in their daily lives, they generally did not perceive themselves as having health problems. For them, health and well-being were transcended by other needs, and physical health and chronic pathologies had low priority. Instead, they said that management of social relationships, emotional health, and stress were central to achieving health and wellbeing.

*“For me, being healthy is being well, being okay with people, with myself and my surroundings, not seeing myself excluded, meaning that people don’t see me and say, “Look, this man is in the street and … damn “It’s disgusting, if he comes, I’ll leave.” (Javier, man, public space)*

*“Tranquility is better than a doctor. I’m not afraid of getting ill. I’m already like “dead”, just as I’m right now, there’s no life. If I’m well organized, I don’t take pills. Because if I work, I will eat better, peace of mind…What does sugar mean? Sugar means nervousness and stress. This means sugar rises.” (Mohamed, man, makeshift shelter)*

### Use and access to the healthcare system

**Figure 1:**
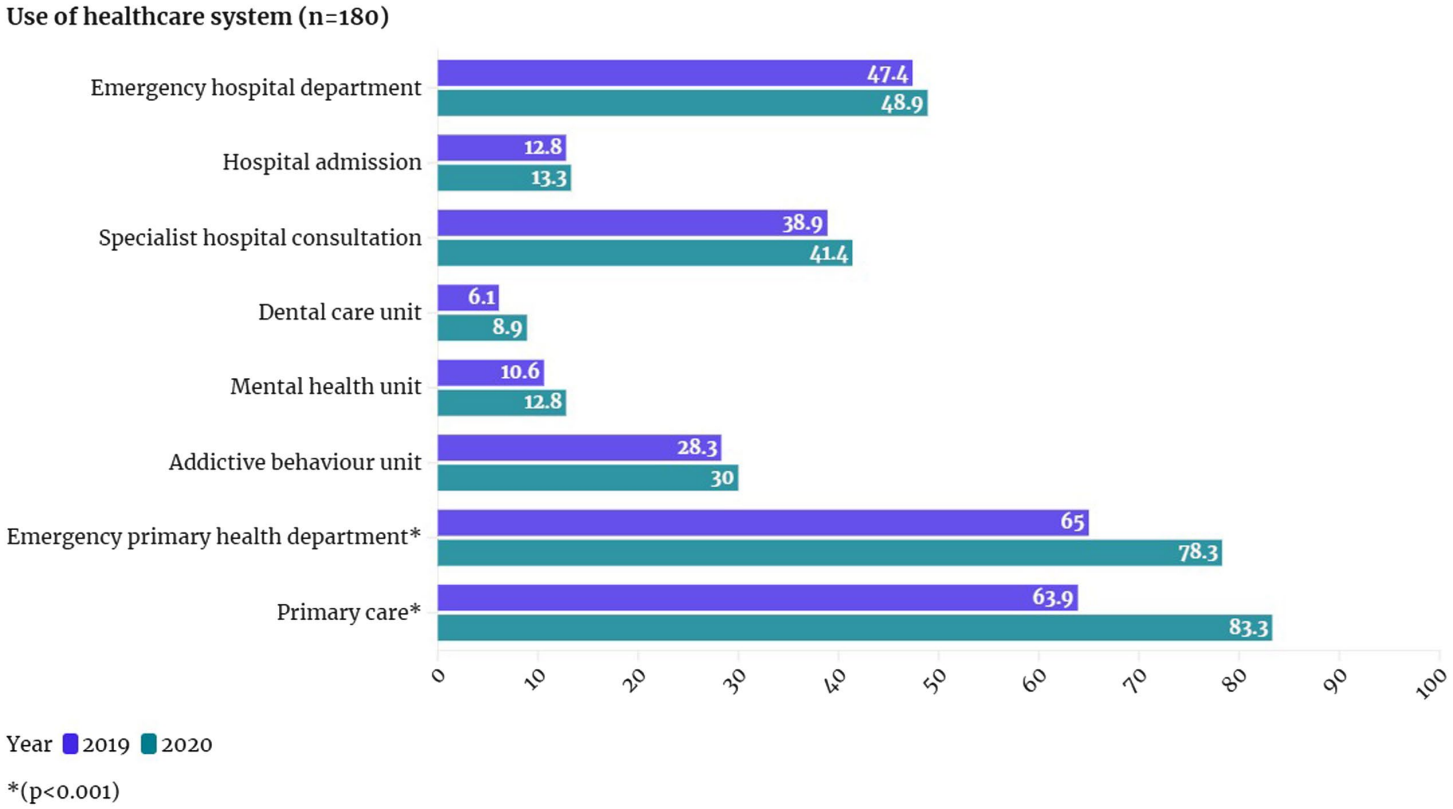
Use and access to the healthcare system.

Nearly all the participants had a health card or a health insurance number (Figure 1), although those who never contacted the system for these documents or were undocumented immigrants did not. The participants reported that barriers to accessing the healthcare system were related to administrative procedures, such as making appointments, obtaining test results, and the general function of the healthcare system, and they sought help from NGOs or social services to overcome these barriers.

*“The first thing I do is go to the doctor, and if he doesn’t listen to me, I go to the social services, and at night, they have to accompany me to the hospital” (Javier, man, public space)*

Primary care was the healthcare service visited by most participants, with utilization rates of 63.9% in 2019 and 83.3% in 2020. Moreover, the participants also frequently visited emergency departments at primary care centers, with rates of 65.0% in 2019 and 78.3% in 2020; this was followed by emergency departments (47.2% in 2019 and 48.9% in 2020) and specialist hospital consultation (38.9% in 2019 and 41.4% in 2020). Something to highlight is that most of them (97.4%) has a sanitary card or ID, and that more than half (60.3%) had a prescription of chronic medication.

The general opinion of the participants was gratitude toward the healthcare system and professionals. At the hospital, they felt comfortable and satisfied with the care provided, and believed they were treated with affection and respect. Participants mainly focused on their experiences during hospital admissions when were asked about this topic. One participant also emphasized that the care provided to him ended the moment he was discharged from the hospital.

*“Do you know where they’ve been good? ‘You are an exceptional patient.’ Until the day they discharged me. What happens after being discharged, it’s not their problem. What matters to them is that you get out of there on your own feet”. (Jose, man, public space)*

### Health needs of HP

The most unsatisfied basic human needs were related to the availability of food, basic hygiene, toilets, and rest, and the avoidance of danger and social isolation (Table 4). The constant search for resources and shelter shaped the daily routine of most participants. This situation created emotional instability that prevented them from establishing a regular routine or achieving a long-term solution that would guarantee fulfillment of their basic needs.

**Table 4:** Basic human needs affected by homelessness.

	Quantitative overall sample *n* (*%*)
Feeding and personal hygiene	
*Nutrition and feeding (n = 188)*	
Public institutions and donations	96 (51.1%)
To do food shopping and donations	76 (40.4%)
Only doing food shopping	16 (8.5%)
No access to drinking water	134 (70.9%)
*IASE (n = 192)*	74 (41.3%)
Unhealthy	49 (25.5%)
Need for change	115 (59.9%)
Healthy feeding	28 (14.6%)
*Dental and chewing problems (n = 188)*	
Pain	57 (29.5%)
Missing teeth	104 (53.9%)
Other problems^a^	12 (6.2%)
*Accessing difficulties to prostheses or assistive devices (n = 119)*	71 (59.7%)
*Usual shower and toilet place (n = 180)*	
Homeless hostel or night shelter	92 (51.2%)
Other places^b^	88 (48.8%)
*Difficulties accessing public toilets (n = 180)*	77 (40.8%)
Sleep, rest and feeling safe	
*Sleeping difficulties (n = 193)* Lack of comfort Related health problems Feelings of fear	89 (46.1%) 19 (9.8%) 61 (31.6%) 9 (4.6%)
*Safety (n = 187)*	
Have suffered some type of assault in the last year	96 (51.3%)
Theft	66 (35.3%)
Physical aggression	33 (17.6%)
Verbal aggression	32 (17.1%)
Extortion	6 (3.2%)
Feeling afraid habitually	61 (32.6%)
Sexual assault *(exclusively women, n = 41)*	5 (12.2%)
Some type of assault *(man n = 146)*	70 (47.9%)
Some type of assault *(woman n = 41)*	26 (63.4%)
Perceived social support	
*Self-perceived social support—SSQ (n = 187)*	
Nobody	53 (28.3%)
One or two people	105 (56.1%)
More than two people	26 (15.5%)

a Tooth loss, gum disease, failing dental work.

b Places such as public toilets, public spaces, bathrooms of friends or relatives, or makeshift shelters.

#### Diet and personal hygiene

According to the IASE measurement of diet quality, most participants have a need to change their diets (59.9%) or have diets with low nutritional value (25.5%). In addition, 53.9% of participants had missing teeth, 29.5% reported dental pain, and 59.7% expressed difficulties accessing prostheses, glasses, or dental prostheses.

Half of the participants relied on formal or informal help from public institutions and citizens to get food, and 40.4% of them shopped for food but also relied on charity for access to food. Many participants (70.9%) reported having limited access to drinking water near their living places and at public overnight shelters.

About half of the participants reported using homeless emergency shelters or hostels as the usual places for showers, and the others reported using public toilets or makeshift shelters to shower using buckets of water or other handmade systems. A small number of participants used the bathrooms of friends or relatives for showers.

*“In shelters you have your three meals a day, you have a shower, social workers give you your shampoo, your towel, they give you your sheets whenever you want, they give you everything man!” (Paco, man, night shelter)*

Despite the risk that these needs would remain unmet, the participants used diverse strategies to meet their basic needs. Specifically, the use of homeless shelters and support from their social environment were perceived as essential.

#### Feeling safe, sleeping, and resting

Almost half of the participants had problems maintaining or falling asleep (Table 4). One-third of them reported this was caused by mood disorders or physical health problems, and the others reported this was related to physical discomfort (9.8%) or being afraid at night (4.6%). In addition, one third of them (32.6%) said they often felt afraid, 51.3% reported being victims of assault, 68.75% were victims of theft, 34.3% were victims of physical aggression, and 33.3% were victims of verbal aggression.

The consequences of the unmet basic human needs adversely impacted the participants’ lives, social relationships, and daily routines, and generated feelings of anxiety and a continuous state of stress.

*“Being able to rest, be calm … Because now you cannot be calm there (abandoned building). Being homeless has affected me a lot (emphasis). A lot, because before, I had friends, we went to buy clothes … now what am I doing here? You cannot move much here. Here, I am surrounded by people you cannot trust much”. (Francisca, woman, abandoned building)*

Moreover, homeless women suffered more assaults than homeless men (63.4% vs. 47.9%); 12.2% of homeless women experienced sexual assault; and 14.6% were diagnosed as victims of gender-based violence while in primary care. This problem is so serious that one woman who was previously in prison said that she preferred being in prison rather than on the streets.

*“But it is tough to live on the street. It is the hardest thing that has happened to me in my life. I prefer jail than living on the street.” (Ana, woman, public space)*

In contrast to feeding and hygiene, participants could not find coping strategies that addressed the need to feel safe and get adequate sleep through social services. The participants who previously or were currently using emergency or night shelters said they felt afraid and insecure while staying there. They also complained about theft and physical aggression.

*“If I were to tell you what happened to me here (shelter) … Everything … €60 has been stolen from me, my wallet with my credit card, with my ID, with the medical card … Last night, they stole all my medication…” (Pedro, man, night shelter)*

#### Perceived social support

The participants had difficulty in maintaining their social environment (Table 4). According to the SSQ, 28.3% of the participants did not have anybody to trust or ask for help, and 56.1% could only rely on one or two people (typically social workers). For those who did have social support, other people provided essential help to cover their needs for food or hygiene. Moreover, even informal social relationships in the neighborhood from local mechanics, librarians, bar owners, waiters, or people who saw them daily, were crucial for maintaining a sense of well-being.

*“There is a man who owns a garage, and one day I was not here, and he called me to see if I was okay, he was worried, and that gives me strength to keep going, you know?” (Javier, man, public space)*

The participants explained that being homeless led them to lose relationships with friends, and even family. Also, being homeless made them lose trust in people, and made it difficult for them to form new relationships.

*“The friends that I had … They all left. They did not want to talk to me if I lived on the street.” (Laura, woman, public space)*

Additional verbatims about all categories supporting the results can be found at Supplementary Table S1.

### Joint display integrating the main results regarding the health of HP

Overall, the results from the quantitative and qualitative analyses were consistent and, in some instances the qualitative results expanded upon the qualitative results. For example, when the quantitative results indicated a relationship between access to the labor market and health, the qualitative results confirmed and expanded upon these findings. However, there were also some notable discrepancies, such as the high prevalence of morbidities in the quantitative analysis and a lack of concern about health issues in the qualitative analysis (Supplementary Table S2).

## Discussion

Our results suggest that structural determinants, particularly the socioeconomic context, were the major determinants of homelessness. Furthermore, homelessness limits the opportunities of HP to attain basic human needs and undermines their health and dignity. Specifically, our findings indicate close interconnections among working conditions, health, labor rights, and housing for individuals experiencing homelessness.

Our results showed that when someone cannot obtain and maintain housing, then the circumstances caused by homelessness lead to an emphasis on survival and fulfillment of basic human needs. These basic needs are focused on food, safety (avoiding dangers and aggression), and shelter for rest, as previously observed (1, 45). To cover these basic needs, HP are often forced to constantly move, and they adopt what could be called a *Nomad-Paleolithic* lifestyle.

This interpretation considers the challenges in classifying HP, because their overnight locations are constantly changing (46). The *Nomad-Paleolithic* lifestyle could increase the morbidity and mortality of HP (47), and also adversely affect their mental health (45). Moreover, the *Nomad-Paleolithic* lifestyle reduces and limits their capabilities, and also decreases the possibility of achieving a minimum level of individual human dignity (25).

The participants in our study show very similar demographic characteristics to other Spanish contexts (48). They also had various physical health problems, such as chronic and infectious diseases (49). In addition, half of them suffered from mood disorders. As previously suggested, elevated levels of anxiety can exacerbate mental and physical health disorders (50). Furthermore, we found that more than half of the participants suffered from episodes of violence and assault (theft, physical violence, verbal threats, and sexual abuse), in agreement with other studies (51). The homeless women in our study were most affected by violence and assault, same as in other studies (52).

Most of our participants visited emergency departments at hospitals or healthcare centers during the two-year study period. Other researchers found that HP tended to use these departments more frequently than the general population (12). The extensive use of these healthcare services by HP could be explained by their higher rates of morbidity and injuries, and their inadequate living conditions. Additionally, the COVID-19 crisis further increased the health risks of HP (53), and likely led to their increased need for healthcare and use of emergency departments during 2019 and 2020. Finally, although the healthcare system of Spain provides universal healthcare coverage, HP are not guaranteed continuous care after discharge from a hospital. Thus, to effectively address health inequities, the healthcare system needs to implement more holistic interventions that prioritize the social aspects of health (54, 55). This approach considers the health and well-being of HP, and our participants reported that emotional health and the social environment were important health-related priorities.

Housing affordability (56), poverty, and social exclusion all affect homelessness (3). Our study was performed in Spain, which adheres to a welfare state model (57), whose foundations are universal healthcare, free public education, protection from unemployment and dependency, and a public pension system (58). Despite these measures, Spain faces structural unemployment and a lack of housing, conditions exacerbated by social and economic crises, such as the COVID-19 pandemic (59). Moreover, Spain is the third-worst country among the EU-27 in terms of people renting at market prices, and on average more than 40% of household income is devoted to housing in Spain (4). This situation shows that although many policies in Spain emphasize the right to adequate housing, its current social welfare model lacks legislation that effectively addresses matters of housing and poverty.

The participants in our study reported that several barriers prevented them from accessing the labor market (long-term unemployment, age of 55 years or more, and lacking higher education), as previously shown (60) to guarantee steady housing, because low-paying jobs do not allow some people to escape poverty or homelessness (61). Other factors that may contribute to homelessness are price inflation, job insecurity, income inequalities, and a poor working environment (4), all of which were identified by our study participants. Moreover, the participants who received social welfare subsidies complained that these benefits were insufficient to guarantee housing, and those who reported receiving no subsidies said there were bureaucratic difficulties in applying for these benefits, as previously reported (62).

Furthermore, as stated by our participants, unemployment has a severe emotional impact because it causes a loss of self-esteem and defines their identity homeless, consistent with other studies (63). This reinforces the assumption that unemployment justifies homelessness (64).

Lastly, in our context, the main intervention for addressing homelessness is a system of shelters. Although shelters provide a place to sleep, eat food, and take showers, many participants said they tended to avoid them because they did not feel safe as shown in other studies (65). They reported preferring to sleep in the open, a behavior leads to maintenance of the *Nomad-Paleolithic* lifestyle (66).

Shelters are the first step of the Spanish public social services system for HP, which is based on the ladder model (67). This model assumes that once people have their basic needs covered (food, rest, and safety) and comply with certain behavioral norms or conditions, then they can achieve economic emancipation only by employment. However, shelters and its hostile environment does not work for some HP and could contribute to perpetuate homelessness (68). Interestingly, given the present system, some participants expressed preferences for being in prison or a hospital, rather than a shelter. By contrast, “Housing First,” a holistic, highly effective, and efficient intervention (69) recently began in Spain (70). This intervention recognizes the importance of providing stable and long-term solutions to people experiencing homelessness, recognizes basic human rights and needs, and prioritizes direct access to permanent housing without preconditions.

Finally, we must point out that the results unexpectedly led us to question the design of our data collection. During the qualitative phase, we observed that the participants considered work and labor conditions, along with survival needs (shelter, safety, and food), to have greater importance and relevance for their health. Although we included these in the quantitative phase, we believe these factors should be prioritized in a qualitative analysis.

## Limitations

Although we tried to reach all of the potentially eligible HP in Palma, our sample selection may have been biased because the HP who participated had some form of contact with social services. Consequently, the HP who did not receive assistance from social services were not represented. A second limitation is that the results should be considered valid only within the specific time frame and environment in which the study was conducted.

## Implications for practice, research, and policy

Policies that aim to eradicate homelessness should consider employment, housing affordability, and the social welfare system, and should also consider the SDOH. Therefore, it is likely that policies which focus on individual aspects, such as the ladder model, will perpetuate or even increase homelessness.

Our study also highlights the relationship between access to the labor market and physical health, especially for those whose income depends on manual labor. These individuals face exclusion when health issues prevent them from working. Therefore, it is essential to provide stronger protections against occupational hazards associated with manual labor and to implement policies that offer alternative work opportunities for these individuals.

The healthcare system should implement an interdisciplinary model that uses a holistic approach to address homelessness. Also, the healthcare system and public and private social organizations should implement health advocacy programs by considering the SDOH and creating a dynamic and opportunities for professionals to implement these programs, with a final goal of contributing to the well-being of everyone.

Our results could serve as a foundation for further analysis of the relationship between housing exclusion, unemployment, and poor health. Longitudinal studies are also needed to examine the consequences of persistent homelessness. In addition, there is a need to evaluate the relationship of gender and homelessness and examine the topics of gender violence and prostitution in this population.

## Conclusion

The health and wellbeing of HP are seriously affected by unemployment and social exclusion. This underscores the relevance of precarious working conditions and the failure of the current welfare system, which may trigger a fall into homelessness or perpetuate homelessness. We found that the main concerns of HP were feeling unsafe and social isolation. We understand that most HP lead a *Nomad-Paleolithic* lifestyle, and this adversely affects their health and increases the risk of premature death. HP are in vulnerable situations and may be denied basic human needs and rights, and many of them cannot attain adequate health-related needs beyond those required for basic survival.

The current system of shelters and other social interventions are insufficient for addressing homelessness because they use paternalistic and individualistic approaches. Moreover, HP perceive shelters as unsafe options that hinder their free will, autonomy, and privacy. Although “Housing First” may provide a better short-term solution, it does not address all of the underlying structural determinants that generate and entrench homelessness.

## Data Availability

The original contributions presented in the study are included in the article/Supplementary material, further inquiries can be directed to the corresponding author.
