# Corrigendum: Health status and self-perception of health among homeless people in Spain: A mixed-methods study

**DOI:** 10.3389/fpubh.2024.1502158

**Published:** 2024-11-27

**Authors:** Miguel A. Bedmar, Laura Capitán-Moyano, Miquel Bennasar-Veny, Cristina Moreno-Mulet, Alba Carrero-Planells, Aina M. Yáñez

**Affiliations:** ^1^Research Group on Global Health and Human Development, University of the Balearic Islands, Palma, Spain; ^2^Department of Nursing and Physiotherapy, University of the Balearic Islands, Palma, Spain; ^3^Health Research Institute of the Balearic Islands (IdISBa), Palma, Spain; ^4^CIBER de Epidemiología y Salud Pública (CIBERESP), Institute of Health Carlos III, Madrid, Spain; ^5^Qualitative and Critical Health Research Group, University of the Balearic Islands, Palma, Spain; ^6^Research Network on Chronicity, Primary Care, and Health Promotion (RICAPPS), Institute of Health Carlos III, Madrid, Spain

**Keywords:** homelessness, social justice, employment, social exclusion, health inequities, social determinants of health

In the published article, there was an error in Figure 1 as published. In the section titled ^*^Emergency Hospital Department^*^: the percentage for the year 2019 is not 97.4, it should be 47.2. The corrected Figure 1 and its caption appear below.

**Figure 1:**
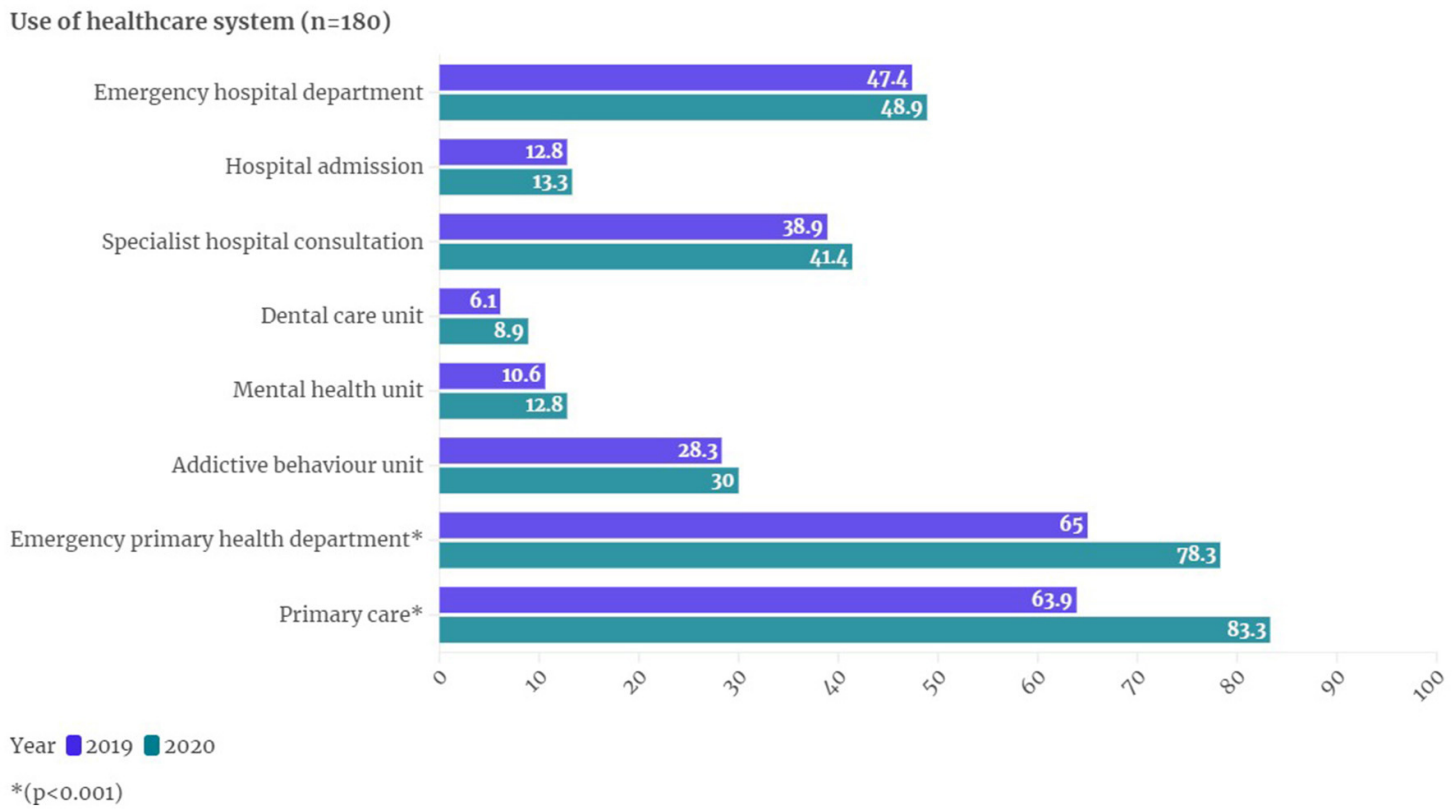
Use and access to the healthcare system.

The authors apologize for this error and state that this does not change the scientific conclusions of the article in any way. The original article has been updated.

