# Supplementary material for: Health status and self-perception of health among homeless people in Spain: a mixed-methods study
Source: Front Public Health. 2024 Aug 29;12:1444888. doi: 10.3389/fpubh.2024.1444888 (PMC11390655; doi:10.3389/fpubh.2024.1444888)
Supplement: Supplementary file 1 [file Data_Sheet_1.DOCX]

Supplementary Material

**Supplementary Table 1.** Additional verbatim.

| Underground economy and unemployment | |
| --- | --- |
| Health problems associated to employment | *“I work in construction, and when I was working, blood came out (rectal hemorrhage), and the doctor told me to not lift heavy weights. I like my job; I’ve done everything, but he (the doctor) tells me, ‘You better quit this job’. It happened before the 2012 economic crisis, and I left construction, but there was no other job. I didn’t know how to find anything else.”* (Emmanuel, man, makeshift shelter)  *“Yes, I used to work; I’ve worked all my life. I’ve worked at the fair setting up the attractions. In recent years, I was a manager and led a couple of people. What happened is that I hurt my back and ... it’s not that I lost it (the job), but I can’t (work). Going to work one day means I will be in pain setting up attractions* *...”* (Javier, man, public space) |
| Difficulties to social and unemployment benefits | *“Phff, they give a lot of problems. Until you are 45-years-old you have no rights, no;* *in theory you have no right to get any social benefit. No way, it has been almost a year. Since last year I haven’t received anything (social benefit). I have been waiting three months for it, and I just got it last month.”* (Antonio, man, abandoned building) |
| Insufficient financial benefits | *“You can’t buy anything, not clothes, anything ... Because with 450€ (per month) or so that the government gives us ... What do I do with that? I can’t even rent a fuc**** flat**.”* (Francisca, woman, abandoned building) |
| Underground economy | *“No, no … in the supermarket, I don’t ask for food. I don’t ask for it. Nor money. What happens is I help them (the clients) parking their cars, and sometimes someone wants to give me something (money).”* (Emmanuel, man, makeshift shelter)  *“I worked so hard since I arrived in Spain, cleaning. Look how my fingers are… (…) Then, in a beach bar in Magaluf (…) I clean the bar, or do the shopping for them, or I run errands for them, or I decorate the local (businesses) for Christmas, and that’s how I earn (money)… Even prostitution”* (Andrea, woman, abandoned building) |
| Emotional impact of unemployment | *“Well, I would also like to have a permanent job, or even if it is temporary, whatever ... But having a job, that is what all normal people do. That I’m no longer normal, I’m subnormal (laughs), I’m already subnormal … (laughs)”* (Francisca, woman, abandoned building)  *“I’ve always had a job ... There have been moments when I haven’t had a job, but I’ve always been a person who has earned a living. No ... I am not someone who stays at his home for a week, with no money and depending on others. I like earning a living, doing things well, working, doing that ...”* (Antonio, man, abandoned building) |

| Health concerns among HP | |
| --- | --- |
| Impact of diseases | *“A lot; it (chronic low back pain) affects me very badly. Very bad, because I feel very handicapped. I can’t go upstairs and downstairs; sometimes, I have been a little hindered. Now I’m a little better, but I have gone so far as to sweep and mop sitting in a chair so I can clean the house.”* (María, woman, abandoned building)  *“And now my hip is bothering me. I would love to be able to get up at six in the morning and run, do some sport. Do you understand me? I went under surgery, but they left me in a bad way, man. They left me badly, man.”* (Pedro, man, night shelter)  *“I’m bad because, of course, between HIV and all that, right? Well, whether you like it or not, I’ve had a very bad time.”*(Antonio, man, abandoned building) |
| Health and well-being for them | *“For me, being healthy is being well, not feeling bad, being good with myself, many things … not having any illness. Being okay with people, not seeing the destruction, that is … being okay with myself and my surroundings, not seeing myself excluded, meaning that people don’t see me and say, “Look, this man is in the street and … damn “It’s disgusting, if he comes, I’ll leave.”* (Javier, man, public space)  *“But what matters most to me is the emotional aspect, because if you have support from a partner or a child who loves you unconditionally ... that you have a shoulder to lean on, that you have...”*(Andrea, woman, abandoned building) |
| Being unhealthy as a consequence of homelessness | *“I like farms. Yes sir, a farm, in an orderly manner. Not like the farm I have in the shack. To work and live. Yes, if it is orderly, you can live there. Better for me. Tranquility is better than a doctor. Hey, really or not? I’m not afraid of getting ill; I’m already like “dead”, just as I’m right now, there’s no life. If I’m well organized, I don’t take pills, I don’t take them. Because to work and a little bit … Do you know? Eat better, peace of mind, that’s it. What does sugar mean? Sugar means nervousness and stress. This means sugar rises.”* (Mohamed, man, makeshift shelter) |

| Use and access to the healthcare system | |
| --- | --- |
| Barriers to accessing to healthcare system | *“No, no, they (primary healthcare center workers) ignore me. No, they don’t listen, no. Here (social services), at least, yes, really. Look, they soon made me glasses, because I said it right here and right away. But if I go to my doctor? My goodness, it would take half a year or more. Half a year or more (emphasis). (...) I find that they (primary healthcare center) have collapsed. They tell you, yes, yes, we’ll call you, we’ll tell you something …”* (Francisca, woman, abandoned building)  *“The first thing I do is go to the doctor, and if he doesn’t listen to me, I go to the UME (social services), and at night, they have to accompany me to the hospital ... they (social services) don’t deny me**.”* (Javier, man, public space) |
| Opinion of healthcare system | *“Look, do you know where they’ve been good? When they treated me, uh, they told me: ‘You are an exceptional patient.’ Until the day they discharged me (…). What happens after being discharged, it’s not their problem. What matters to them is that you get out of there on your own feet”.* (Jose, man, public space)  *“The hospital, for me, is great. I did not want to go back (to the street).”* (Andrea, woman, abandoned building) |

| Health needs of HP | |
| --- | --- |
| Constant research of resources | *"There are 24 hours in a day, and ... where do you seek refuge? Maybe, due to the necessity of moving around, you get caught in the rain, and then you have to change your clothes. What do you do? Well, I go anywhere, and I change as best as I can ..."* (Miguel, man, public space) |
| Diet and personal hygiene | *"What if today I do not have anything to eat? Well, I am going to Sa Placeta or Ca l'Ardiaca (emergency shelters). I am allowed to go there to eat when I need it."* (Antonio, man, abandoned building)  *“I am going to get water at my son’s house. Moreover, I shower at my partner’s or son’s house. I cannot shower every day, but I shower every other day ... Or at my Colombian friend’s house, who loves me very much.”* (Andrea, woman, abandoned building)  *“This (showering) is a privilege that others do not have because they do not want to, man. I see you have your three meals a day, you have a shower, they (shelter workers) give you your shampoo, your towel, they give you your sheets whenever you want, they give you everything man!”* (Paco, man, night shelter) |
| Feeling safe, sleeping, and resting | *“Because having comforts is very lovely, huh? Being able to rest, be calm ... Because now you cannot be calm there (abandoned building). Then one day, they (other people who live there) set your house on fire, and you do not know how to get out in a hurry. That’s what happened to us yesterday or the day before yesterday ... Of course, this situation (being homeless) has affected me a lot (emphasis). A lot, because before, I had friends, we went to buy clothes ... now what am I doing here? You cannot move much here. Here, I am surrounded by people you cannot trust much”.*(Francisca, woman, abandoned building)  *“When I sleep, I am a little insecure; I do not sleep, I rest, but I do not sleep, and I doubt if anyone on the street sleeps. Not because they rob you, but because they can attack you or whatever ... The first month has been disgusting, then there comes a point where you get used to it**.”* (Javier, man, public space)  *“He (the aggressor) covered my mouth in the park when I was going towards the old prison (abandoned building). He followed me, sat me down, and told me: “Baby, baby, sit down for a little while.” He started touching me … Thank goodness J (a friend) came ... I said: “J!” Then he took him aside, and I do not know what he must have done to him, but he has never bothered me again, nor have I ever seen him again.”* (Andrea, woman, abandoned building)  *“But it is tough to live on the street. It is the hardest thing that has happened to me in my life. I prefer jail than living on the street.”* (Laura, woman, public space)  *“If I had a room for myself (he must be sharing a room with six or eight people), I could go there and shower, not listen to anyone’s snoring, not smell anyone’s feet ... Leave my cell phone on the bed and not have it taken from me ... Because this (sleeping in the shelter) is something else, you can’t leave anything, not even dirty socks, because someone else puts them on, so I do not go there now.”* (Jose, man, public space)  *“There is something else, fear, terror ... Here, (in the shelter) to be robbed ... Here there are some very crazy people, man! Imagine that you are asleep, and a madman takes a knife and starts stabbing someone, goes into a room and starts stabbing people. It is possible!”*(Sergio, man, night shelter)  *“If I were to tell you what happened to me here … Everything… €60 has been stolen from me, my wallet with my credit card, with my ID, with the medical card … Last night, they stole all my medication. They left me without medication ...”*(Pedro, man, night shelter) |
| Perceived social support | *“There is a man who owns a garage, and one day I was not here, and he called me to see if I was okay, he was worried, and that gives me strength to keep going, you know?”*(Javier, man, public space)  *“People I have met walking down the street have helped me more than those who have known me for 20 years. It is strange to say it, but it is true (emphasis).”* (Jose, man, public space)  *“The friends that I had … They all left. They did not want to talk to me if I lived on the street.” (Laura, woman, public space)* |

**Supplementary Table 2.** Joint display integrating the main results regarding the health of HP.

| Joint display of the integrated quantitative and qualitative data | Outcomes |
| --- | --- |
| 1. 91.4% of our participants were unemployed. The most-mentioned barriers to accessing the labor market were health issues (27.3%).   *“Yes, I used to work. I’ve worked at the fair setting up the attractions. What happened is that I hurt my back and ... it’s not that I lost it (the job), but I can’t (work). Going to work one day means I will be in pain setting up attractions ...”* (Javier, man, public space) | Confirmation/Expansion |
| 1. Close than 70% of unemployed HP did not receive any social welfare benefits. Those who received some subsidies were still on the streets.   *“You can’t buy anything, not clothes, anything ... Because with 450€ (per month) ... What do I do with that? I can’t even rent a fuc**** flat.”* (Francisca, woman, abandoned building) | Expansion |
| 1. Lack of employment was not explored as a possible factor to consider in the HP’s health and dignity.   *“I’ve always been a person who has earned a living. I am not someone who stays at his home for a week, with no money and depending on others. I like earning a living, doing things well, working.”* (Antonio, man, abandoned building) | Expansion /  Discordance |
| 1. Approximately half of the HP were diagnosed with one or more chronic diseases. Also, more than half of the HP were also diagnosed with a mental health condition. However, HP assigned a low priority to physical health or the development of their chronic pathologies in their global health.   *“What matters most to me is the emotional aspect, because if you have support from a partner or a child who loves you unconditionally ...”* (Andrea, woman, abandoned building) | Discordance |
| 1. Nearly all the participants had sanitary cards and access to the healthcare system. Nevertheless, participants referred to barriers to accessing the healthcare system related to administrative processes such as making appointments and obtaining test results.   *" The healthcare center workers ignore me. They tell you, yes, yes, we'll call you, we'll tell you something… Social services, at least, yes. Look, they soon made me glasses, but if I go to my doctor? My goodness, it would take half a year or more…* (Francisca, woman, abandoned building) | Discordance |
| 1. The general opinion of the participants was gratitude toward the healthcare system and healthcare professionals. Participants focused on their experiences during their hospital admissions.   *“The hospital, for me, is great. I did not want to go back (to the street).”* (Andrea, woman, abandoned building) | Confirmation/Expansion |
| 1. Close to 60% of our participants needed to change their diet, and more than half relied on formal help from public institutions to get food. Also, more than 70% did not have access to drinking water, showers, or toilets close to them. Feeding and hygiene were at risk; however, HP had coping strategies to cover these needs.   *"What if today I do not have anything to eat? Well, I am going to shelters when I need to eat."* (Antonio, man, abandoned building) | Expansion/  Discordance |
| 1. Almost half of HP had problems maintaining or falling asleep. HP mainly related this problem to mood disorders or physical health problems. In addition, one-third of them often feel afraid, and more than half suffered some type of assault during the past year. Scoping strategies were not enough to cover these needs.   *"There is something else, fear, terror… Here there are some very crazy people, man! Imagine that you are asleep, and a madman takes a knife and starts stabbing someone, goes into a room, and starts stabbing people. It is possible!"* (Sergio, man, night shelter) | Confirmation/  Expansion |
| 1. The social environment was also at risk. Close to 30% of HP had nobody they could trust or ask for help, according to SSQ.   *“None, friends, none. I don’t have a friend’s trust. I don’t trust them, man. My fellow countrymen are very fu**ed up (emphasis), everyone. They steal, they fu** around ... I don’t like it.”*(Mohamed, man, makeshift shelter) | Confirmation/Expansion |
